# Evaluation of Toxic Metals and Essential Elements in Children with Learning Disabilities from a Rural Area of Southern Brazil

**DOI:** 10.3390/ijerph111010806

**Published:** 2014-10-17

**Authors:** Sabrina Nunes do Nascimento, Mariele Feiffer Charão, Angela Maria Moro, Miguel Roehrs, Clovis Paniz, Marília Baierle, Natália Brucker, Adriana Gioda, Fernando Barbosa, Denise Bohrer, Daiana Silva Ávila, Solange Cristina Garcia

**Affiliations:** 1 Laboratory of Toxicology (LATOX), Department of Analysis, Federal University of Rio Grande do Sul, Porto Alegre, RS 90610000, Brazil; E-Mails: sabrinascimento@hotmail.com (S.N.); marifeiffercharao@yahoo.com.br (M.C.); angelammoro@yahoo.com.br (A.M.); miguelroehrs@yahoo.com.br (M.R.); clovis.paniz@yahoo.com.br (C.P.); mariliabaierle@yahoo.com.br (M.B.); nataliafarma@hotmail.com (N.B.); 2 Post-Graduate Program in Pharmaceutical Sciences (PPGCF), Federal University of Rio Grande do Sul, Porto Alegre, RS 90610000, Brazil; 3 Faculty of Pharmacy, University of Caxias do Sul, Caxias do Sul, RS 95070560, Brazil; 4 Department of Clinical and Toxicology Analysis, Federal University of Santa Maria, Santa Maria, RS 97119900, Brazil; 5 Department of Chemistry, Pontifical Catholic University of Rio de Janeiro (PUC-Rio), Rio de Janeiro, RJ 22451900, Brazil; E-Mail: agioda@hotmail.com; 6 Laboratory of Toxicology and Essentiality of Metals, Faculty of Pharmaceutical Sciences of Ribeirão Preto, University of São Paulo, Ribeirão Preto, SP 14040903, Brazil; E-Mail: fbarbosa@fcfrp.usp.br; 7 Chemistry Department, Federal University of Santa Maria, Santa Maria, RS 97105900, Brazil; E-Mail: bohrer.denise@gmail.com; 8 Post-Graduate Program in Biochemistry, Federal University of Pampa, Uruguaiana, RS 97500970, Brazil; E-Mail: avilads1@gmail.com

**Keywords:** rural children, essential and toxic elements, cognitive ability, ALA-D inhibition, oxidative stress

## Abstract

Children’s exposure to metals can result in adverse effects such as cognitive function impairments. This study aimed to evaluate some toxic metals and levels of essential trace elements in blood, hair, and drinking water in children from a rural area of Southern Brazil. Cognitive ability and δ-aminolevulinate dehydratase (ALA-D) activity were evaluated. Oxidative stress was evaluated as a main mechanism of metal toxicity, through the quantification of malondialdehyde (MDA) levels. This study included 20 children from a rural area and 20 children from an urban area. Our findings demonstrated increase in blood lead (Pb) levels (BLLs). Also, increased levels of nickel (Ni) in blood and increase of aluminum (Al) levels in hair and drinking water in rural children were found. Deficiency in selenium (Se) levels was observed in rural children as well. Rural children with visual-motor immaturity presented Pb levels in hair significantly increased in relation to rural children without visual-motor immaturity (*p* < 0.05). Negative correlations between BLLs and ALA-D activity and positive correlations between BLLs and ALA-RE activity were observed. MDA was significantly higher in rural compared to urban children (*p* < 0.05). Our findings suggest that rural children were co-exposed to toxic metals, especially Al, Pb and Ni. Moreover, a slight deficiency of Se was observed. Low performance on cognitive ability tests and ALA-D inhibition can be related to metal exposure in rural children. Oxidative stress was suggested as a main toxicological mechanism involved in metal exposure.

## 1. Introduction

Children’s brain development is susceptible to damage resulting from sustained exposure to harmful environmental factors. In comparison to an adult, the brain of a child is more vulnerable to injury caused by toxic agents [1]. Additionally, children are growing and developing and when exposed to chemicals at critical stages in their cognitive and physical developments it may have serious consequences. The effects of environmental chemicals on children’s health have been reported extensively, with the majority focusing on the adverse effects on the central nervous system (CNS) [1,2,3,4,5,6].Furthermore, children’s hand to mouth behavior as well as playing close to the ground also increases their probability of exposure [7,8,9].

Recently, several studies have demonstrated adverse effects on children’s health related to the exposure to metal, where the main consequences were intelligence and attention deficits [5,10,11,12]. Metal exposure during the development period can lead to permanent behavioral, developmental and functional impairments [10]. These chemicals occur naturally in the environment and children’s exposure may occur by anthropogenic sources, such as agriculture practices [13]. Because of this, children living in rural areas can be exposed to metals resulting, for example, from farm machinery and from the use of fungicides, insecticides and herbicides [14]. 

Lead (Pb) is one of the most well-known metals to affect the CNS, with chronic Pb exposure associated with impairments to physical growth, learning, memory, and leading to cognitive, behavioral and psychological disorders in children [4,15,16,17]. Despite the neurotoxicity of Pb having been well established throughout history, recently, there has been a growing concern about “safe” limits of Pb exposure. Recent studies have demonstrated an association between Pb blood levels below 10 µg·dL^−1^ and reduced IQ and cognitive functions deficits, learning difficulties and impaired growth in children [18,19,20,21]. However, some researchers even argue that any concentration of Pb in the body can lead to dysfunction of biochemical processes in the brain [13,22,23]. Additionally, cognitive, behavioral, and neuropsychological effects were also related to children’s exposure to other metals, such as arsenic (As), cadmium (Cd), manganese (Mn), and mercury (Hg) [24,25,26]. Also, in some cases, drinking water is considered to be the main source of metal exposure [26]. Moreover, most environmental exposures to metals do not occur in an isolated manner. Thus, it is important to study the adverse effects on health of affected populations to multiple metals exposure [5].

Additionally, biomonitoring of metals considered essential in human biologic samples is very important for occupational and environmental health, mainly because metals are required for normal physiological function and are involved in numerous biochemical mechanisms [27]. Moreover, when at higher concentrations than those required for biologic functions in the body, these essential trace elements can be toxic [28,29].

Furthermore, several specimen matrices are available for toxic and essential trace element quantifications, including blood, serum/plasma, urine, saliva, hair, and nails. Although whole blood is the specimen most used, hair has been considered as a promising biological specimen for analysis of routine clinical screening of toxic metals exposure and essential trace elements status in the human body [30]. However, the choice of the biological sample depends on factors such as toxicokinetics and collection procedures. The hair reflects long term exposure and has been more accepted by children than blood as collection causes no pain or other nuisances. Also, hair is non-invasively collected, easily stored, and readily transported to the laboratory for analysis [27,31,32]. Moreover, some metals have high affinity for sulfhydryl (-SH) groups mainly in keratinized tissues, such as hair [32].

Additionally, oxidative stress is considered the main mechanism involved in the pathophysiology of metal intoxication, mainly due to Pb and aluminum (Al) [22,33]. The oxidative stress consists of a state where the antioxidant defense is disturbed by an increase in radical formation leading to oxidative damage to biomolecules [34]. An important biomarker of oxidative stress is malondialdehyde (MDA), a secondary product of lipid peroxidation which can be used as an indicator of cell membrane injury [35].

Besides, an important enzyme to be evaluated when exposure to metals is investigated, is the δ-aminolevulinate dehydratase (ALA-D). ALA-D is involved in the heme biosynthesis pathway catalyzing the condensation of two aminolevulinic acid (ALA) molecules to produce porphobilinogen, which is the precursor of the heme group of hemoglobin [36]. Additionally, ALA-D is a zinc metalloenzyme possessing thiol (-SH) groups, which are essential for its activity [36,37]. Furthermore, ALA-D have high sensitivity to -SH oxidation by pro-oxidant elements, such as toxic metals, leading to inhibition of their activity [36,38]. For Pb exposure, the ALA-D activity has been considered an important clinical biomarker for a long time, since Pb binds to -SH groups inhibiting this enzyme activity [22,36,39,40].

In this respect, the main objective of this study was to evaluate the levels of some toxic metals—aluminum (Al), arsenic (As), cadmium (Cd), lead (Pb), mercury (Hg) and nickel (Ni)—and essential trace elements—cobalt (Co), copper (Cu), manganese (Mn) and selenium (Se)—in biological samples, together with cognitive ability and biomarkers of oxidative stress (ALA-D and MDA) in children.

## 2. Methods 

### 2.1. Study Population

Children for this study were selected from two areas of the central region of Rio Grande do Sul, Southern Brazil. The first group was composed of twenty school-aged children (8–14 years; nine girls and 11 boys), living in a rural area (rural children) of a small city with approximately 17,000 inhabitants characterized by agricultural activities. These children presented learning disabilities, according to teachers of their school. Thus, this study can be considered a query of public health.

Since there are no reference values for the oxidative stress biomarkers performed in this study, a control group consisting of twenty school-aged children (8–14 years; 10 girls and 10 boys) living in an urban zone (urban children), which contain approximately 260,000 inhabitants characterized to be an academic town, was used for comparison.

The study was approved by the Ethics Committee for Research of the Santa Maria Federal University/RS (CAAE 0147.0.243.000-06). Written informed consent was obtained from children’s parents in all instances after given full explanation of the study. 

### 2.2. Cognitive Ability Assessment

The cognitive ability was assessed in all 20 children from rural areas, and one child was excluded due to a previous diagnosis of attention-deficit hyperactivity disorder (ADHD). Thus, 19 children were included in the assessment. Two cognitive tests were used to evaluate the cognitive ability, which were administered by a trained psychologist. The completion of the cognitive tests occurred during school hours in rooms provided by the school in which the participants were linked. First, the Bender Test for visual motor skills was used. This test consisted of nine figures that were separately presented to children who had to copy them as best as they could on a blank sheet [41], and as a result the children were classified as presenting with or without visual motor immaturity. Secondly, the R-2 Intelligence Test was applied as a nonverbal intelligence test for intellectual assessment of the children. The test consisted of 30 blank or colored cards, to be applied in the sequence of their numbers, with images of concrete objects or abstract images. Each figure was displayed to the child with a missing part, and the child was oriented to indicate, among the available alternatives, the alternative which correctly completed the drawing. The answers were recorded by the investigator and the test was applied individually without time limit [42]. Results from the R-2 Intelligence Test were categorized into six different subgroups, according to degree of intelligence as follows: upper average (Group I), above-average (Group II), average intelligence (Group III), lower average (Group IV), borderline (Group V) or poor intelligence (Group VI), respectively.

### 2.3. Sample Collection

Blood samples from all children were collected by routine arm venous punctures into three Vacutainer^®^ (BD Diagnostics, Plymouth, UK) tubes containing (a) EDTA (Trace Metal Free), (b) sodium heparin and (c) no anticoagulants. An EDTA-blood tube (4 mL) was used for hemoglobin and hematocrit measurements; and for trace element quantifications. The remaining blood was immediately centrifuged at 1500*g* for 10 min at 4 °C, and plasma was used to quantify MDA. The heparin sodium-blood tube (4 mL) was collected and stored at –80 °C and analyzed to determineALA-D activity and the index of reactivation of ALA-D (ALA-RE). Serum samples were obtained by centrifugation of blood without anticoagulants at 1500*g* for 10 min at 4 °C for creatinine, urea and for the hepatic enzymes aspartate aminotransferase (AST), alanine aminotransfe­rase (ALT) and gamma-glutamyl transferase (GGT), in order to establish kidney and liver function, respectively. 

Hair samples were collected with surgical stainless steel scissors, by removing a 3-cm-strand of hair, weighing between 250 and 500 mg from the occipital region, just above the neck; and were used to analyze trace element concentrations in 11 out of 20 children of the rural children. Each child’s name and date of sample collection was identified and then stored in polyethylene bags.

Also, approximately 10 mL of a drinking water sample (tap water) from the kitchen of each child’s household was collected. The tap water of rural children’s households comes from shallow wells and cement-made wells. Drinking water samples were transferred to tubes previously acidified with 100 µL of ultrapure nitric acid. Tubes were properly identified and stored at 4 °C until determination of metal levels. The samples were identified and stored in sterilized polyethylene bottles.

### 2.4. Hematological and Biochemical Analyses

Hematological and biochemical parameters were evaluated only in rural children. Hemoglobin (Hb) and hematocrit (Ht) were determined in the ABX Pentra 80 (Hematology Analyzer—Diamond Diagnostics, San Francisco, CA, USA). The serum biochemical parameters evaluated were creatinine and urea for kidney function and AST, ALT, and GGT for liver function. Biochemical analyses were determined by humid chemistry using the device Cobas Integra 400 Plus® (Roche Diagnostics, Indianapolis, IN, USA). 

### 2.5. Toxic Metals and Essential Trace Elements in Blood

Quantification of toxic metals and essential trace elements in whole blood was performed on rural children. The toxic metals quantified were: As, Cd, Ni, and Pb. The essential trace elements quantified were: Co, Cu, Mn, and Se. The trace element concentrations were assessed as previously described by inductively coupled plasma mass spectrometry (ICP-MS; Elan DRCII, PerkinElmer, Sciex, Norwalk, CT, USA) [43,44]. All reagents used were of analytical grade. Internal standard rhodium (1000 mg·L^−1^) and the multi-element (10 mg·L^−1^) solution were obtained from PerkinElmer (Shelton, CT, USA). Samples were diluted at 1:50 with 0.01% (v/v) Triton® X-100 and 0.5% (v/v) sub-distilled nitric acid. Each curve calibration point (blank, reagent blank and sample) was analyzed with 10 mg·L^−1^ of internal standard rhodium. The limits of detection (LOD) for metals were: Cd (0.04 µg·L^−1^); Co (0.11 µg·L^−1^); Cu (0.05 µg·dL^−1^); Mn (0.09 µg·L^−1^); Ni (0.12 µg·L^−1^); Pb (0.004 µg·L^−1^); Se (0.1 µg·L^−1^).

### 2.6. Toxic Metals and Essential Trace Elements in Hair

Quantification of toxic metals and essential trace elements in hair was performed on rural children. The toxic metals quantified were: Al, As, Cd, Hg, Ni, and Pb. The essential trace elements quantified were: Co, Cu, and Se. The analysis was performed by ICP-MS after hair washing, acid digestion and subsequent determination of the due elements [45]. First, hair was successively washed with acetone/EXTRAN^® ^(1% v/v)/Milli-Q water in an ultrasonic bath to eliminate exogenous elements. After, a digestion process (overnight at room temperature) was performed on hair samples, where 2.50 mL of HNO_3_ (sub-bidistilled) was added for each 0.25 g of sample in a 50-mL-polypropylene tube and finally, the process was followed by a further 2h-digestion blocker at 80 °C. Then, 1 mL of peridrol was added and samples were heated for a further 15 min. Subsequently, samples were diluted to a final volume of 25 mL and metals were determined by ICP-MS as mentioned above. The LODs for metals were (µg·g^−1^): As (0.04); Cd (0.006); Co (0.001); Hg (0.005); Ni (0.01); and Pb (0.003); Se (0.05). 

### 2.7. Toxic Metals in Drinking Water

Analyses of trace elements in drinking water were carried out immediately at the home of the child who underwent hair analysis; in order to verify whether water could be a source of contamination. The population of the rural area uses drinking water from shallow wells. Only toxic metals were quantified in drinking water: Al, As, Cd, Hg, Ni, and Pb. The analysis was performed by ICP-MS. Calibration was performed using standard solutions at concentrations of 1 mg·L^−1^ (Perkin Elmer 29 and Merck Titrisol) and acidified with bi-distilled nitric acid. Calibration curve concentrations ranged from 10–100 mg·L^−1^, and the internal standard used was Rh at a concentration of 10 mg·L^−1^. The limit of detection (LOD) and limit of quantification (LOQ) were calculated using the equationLOD= 3 × SD/S and LOQ= 10 × SD/S, respectively, where SD represents the standard deviation of the readings of 10 blanks and S is the sensitivity of the analytical curve (slope). The metal concentrations in drinking water were expressed in mg·L^−1^. The LODs for metals were (mg·L^−1^): As (0.00008); Cd (0.00004); Ni (0.00012); and Pb (0.0004).

### 2.8. Quantification of Plasmatic MDA Levels

Lipid peroxidation was determined by measurement of MDA levels in plasma of both children’s groups. Plasma MDA was analyzed by high performance liquid chromatography with a visible detector (HPLC-VIS), as described previously [35].

### 2.9. Blood δ-Aminolevulinate Dehydratase (ALA-D) Activity 

ALA-D activity was assayed in whole blood samples of both children’s groups, according to the method described previously with minor changes [46]. The enzymatic activity was determined by the rate of porphobilinogen (PBG) formation, in the presence and absence of the reduction agent dithiothreitol (DTT 2 mM). After 10 min. of pre-incubation, the enzymatic reaction was started by adding the substrate ALA 4 mM in PBS at pH 6.8. Incubation was carried out for 1h at 37 °C and reaction product was measured at 555 nm. The ALA-D reactivation index (ALA-RE) was estimated using the equation: A – B/A × 100, where A is the absorbance of ALA-D with DTT and B is the absorbance of ALA-D without DTT. 

### 2.10. Statistical Analysis

Analysis of data was performed using IBM SPSS software version 19.0 (IBM Corp., Armonk, NY, USA). All the study variables were tested for normality by the Shapiro-Wilk test. Statistical analyses were performed using student’s *t*-test to compare the means between groups with normal distribution. The Mann-Whitney test was used for variables with non-normal distribution to verify statistical differences between groups. The results were expressed as mean ± standard error of the mean (SEM) or median (interquartile range), according to distribution of variables. Spearman’s rank correlation analysis was carried out to evaluate the associations between pairs of variables. Statistical significance was considered when *p* < 0.05. 

## 3. Results 

As shown in Table 1, rural children showed no change in hematologic parameters such as Hb and Ht. Also, no changes were observed concerning hepatic and renal functions, which were within the reference values [47,48]. 

**Table 1:** Hematological and biochemical parameters in rural children (*n* = 20).

Parameters	Rural Children	Reference Values *
Hb (g·dL^−1^)	13.24 ± 0.17	12.0–16.0
Ht (%)	39.64 ± 0.45	34–45
AST (U·L^−1^)	24.30 ± 1.05	10–40
ALT (U·L^−1^)	15.25 ± 1.06	10–35
GGT (U·L^−1^)	9.0 ± 0.51	≤50
Urea (mg·dL^−1^)	23.02 ± 1.31	11–39
Creatinine (mg·dL^−1^)	0.36 ± 0.02	0.3–0.7

Notes: The values are expressed as mean ± standard error of the mean (SEM). AST: aspartate aminotransferase; ALT: alanine aminotransferase; GGT: gamma-glutamyl transferase. * Burtis and Ashwood [47,48].

Concentrations of the toxic metals and essential trace elements in blood samples of rural children are shown in Table 2. Regarding the toxic metals, the blood Pb levels (BLLs) were higher than recommended by World Health Organization (WHO) [49]. Nickel levels were also increased in relation to recommended by WHO. Moreover, Cd levels were slightly lower than reference values. In relation to essential trace elements, the results demonstrated that the Se levels were slightly lower than referenced. 

**Table 2:** Concentrations of toxic metals and essential trace elements in blood of rural children (*n* = 20).

Elements	Rural Children	WHO Desirable/Tolerable Range *
*Toxic metals*		
As (μg·L^−1^)	3.95 ± 0.08	2.0–20.0
Cd (μg·L^−1^)	0.04 ± 0.01	0.3–1.2
Pb (μg·dL^−1^)	42.06 ± 8.71	5–15
Ni (μg·L^−1^)	4.65 ± 1.39	<1.0
*Essential elements*		
Co (μg·L^−1^)	0.19 ± 0.02	0.1–0.3
Cu (μg·dL^−1^)	102.7 ± 25.56	80–111
Mn (μg·L^−1^)	11.21 ± 0.53	8.0–12.0
Se (μg·L^−1^)	72.33 ± 2.342	75–120

Notes: The values are expressed as mean ± standard error of the mean (SEM); * WHO, 1996 [49].

Table 3 shows the concentrations of the toxic metals and essential trace elements in hair of rural children. Among toxic metals, only Al levels in hair were greater than the reference values. The Co levels were higher than reference values established for hair of the Brazilian population [45]. With regard to Se, deficiency was also observed in hair samples with levels lower than the reference values. 

**Table 3:** Concentrations of toxic metals and essential trace elements in hair of rural children (*n* = 11).

Elements (µg·g^−1^)	Rural Children (µg·g^−1^)	Reference Values for Human Hair (µg·g^−1^) *
*Toxic metals*		
Al	52.0 ± 9.0	<14
As	0.04 ± 0.008	<0.15
Cd	0.28 ± 0.09	<0.3
Hg	0.19 ± 0.03	<2.3
Pb	1.46 ± 0.27	<9.3
Ni	0.08 ± 0.008	<0.6
*Essential elements*		
Co	0.06 ± 0.01	0.003–0.03
Cu	12.88 ± 2.50	10–32
Se	0.65 ± 0.05	0.8–1.5

Notes: The values are expressed as mean ± standard error of the mean (SEM); * Miekeley* et al.* 1998 [45].

Analysis of toxic metal concentrations in drinking water from households of rural children showed high concentrations of Al, above the acceptable limits by WHO [50]. The results are shown in Table 4.

**Figure 1:**
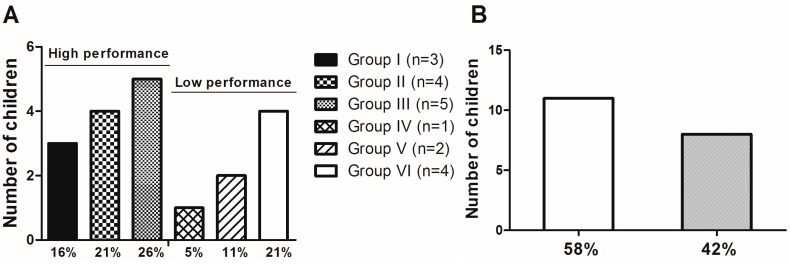
Results from the cognitive function assessment in rural children (*n* = 19). (**A**) R-2 Intelligence Test (*n* = 19). Children were categorized into six different groups: Group I—upper average (16%, *n* = 3); Group II—above-average (21%, *n* = 4); Group III—average intelligence (26%, *n* = 5); Group IV – lower average (5%, *n* = 1); Group V—borderline (11%, *n* = 2); Group VI—poor intelligence (21%, *n* =4). (**B**) Bender Test. Children were categorized into two groups: Group I—children with visual-motor immaturity (58%, *n* = 11); Group II—children without visual-motor immaturity (42%, *n* = 8).

**Figure 2:**
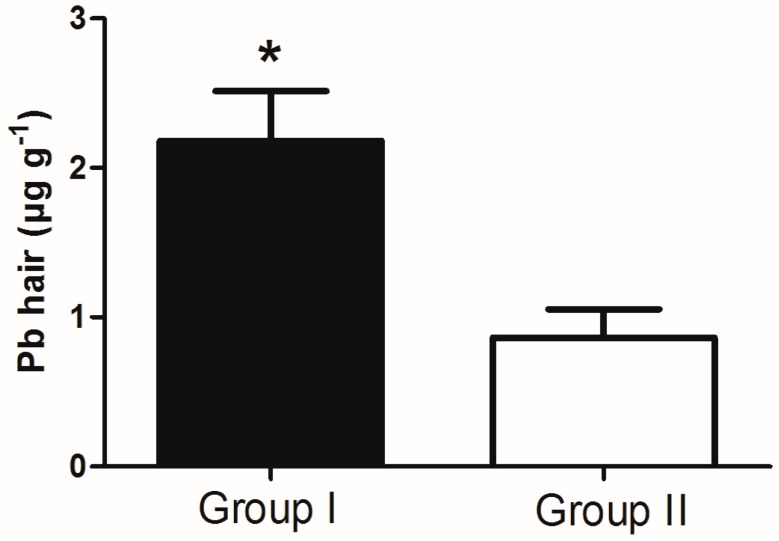
Rural children were divided into two groups according to different results in the Bender Test and significant differences between concentrations of Pb in hair: Group I—children with visual-motor immaturity (*n* = 5) and Group II—children without visual-motor immaturity (*n* = 6); * *p* < 0.01.

Cognitive ability assessment was performed in all rural children using two neuropsychological tests: R-2 intelligence test and Bender test. Approximately 37% of rural children demonstrated low performance in the R-2 intelligence test (Figure 1A) and 58% of rural children demonstrated visual-motor immaturity in the Bender test (Figure 2A).

**Table 4:** Concentrations of toxic metals in drinking water from the households of rural children (*n* = 11).

Toxic Metals (mg·L^−1^)	Drinking-Water Samples (mg·L^−1^)	WHO Desirable/Tolerable Range (mg·L^−1^) *
Al	0.4 ± 0.08	0.1
As	0.001	0.01
Cd	0.001	0.003
Hg	0.002 ± 0.0002	0.006
Ni	0.002 ± 0.0007	0.07
Pb	0.002 ± 0.0002	0.01

Notes: The values are expressed as mean ± standard error of the mean (SEM); * WHO, 2011 [50].

Moreover, the levels of Pb in hair of rural children with visual-motor immaturity (Group I) were significantly increased in comparison to rural children without visual-motor immaturity (Group II), according to Figure 2 (*p* < 0.01).

Table 5 demonstrates the results of the biomarkers of oxidative stress. The results of the lipid peroxidation assessed by MDA measurement demonstrated that rural children had levels significantly increased of plasmatic MDA levels in comparison to urban children, being 6.50 ± 0.18 µmol·L^−1^ *vs.* 3.85 ± 0.19 µmol·L^−1^, respectively (*p* < 0.001). Blood ALA-D activity showed no significant differences between the children groups (*p* > 0.05). Additionally, the involvement of sulfhydryl groups in the ALA-D inhibition was examined by testing the effect of DTT on the enzyme in both children’s groups. The ALA-RE activity was assessed by the addition of DTT (2 mM) to the assay mixture, which caused an increase of 54.91 ± 10.55% *vs.* 26.65 ± 3.72% in the enzymatic activities of the rural and urban children, respectively (*p* < 0.05). 

Additionally, Spearman’s rank correlation showed a significant tendency that the increase of BLLs was accompanied by a decrease of ALA-D activity (*r* = −0.605; *p* = 0.06; *n* = 20). Moreover, a positive Spearman’s rank correlation was found between ALA-RE activity and BLLs (*r =* 0.693; *p =* 0.001; *n =* 20). 

**Table 5:** Biomarkers of oxidative stress in rural and urban children.

Biomarkers	Rural Children (*n* = 20)		Urban Children (*n* = 20)		*p* value
	Mean ± SEM	Median (*Q*_25-75_)	Mean ± SEM	Median (*Q*_25-75_)	
MDA (µmol·L^−1^)	6.50 ± 0.18	6.34 (5.8–6.82)	3.85 ± 0.19	3.87 (3.40–4.33)	<0.001 ^a^
δ-ALA-D (U·L^−1^)	21.42 ± 1.65	22.31 (15.16–28.43)	21.33 ± 1.19	22.19 (17.07–24.94)	0.097 **^b^**
δ-ALA-RE (%)	54.91 ± 10.55	49.05 (19.66–76.56)	26.65 ± 3.72	24.24(15.53–43.23)	0.023 **^a^**

Notes: MDA: malondialdehyde; δ-ALA-D: δ-aminolevulinate dehydratase; δ-ALA-RE: δ-ALA-D reactivation index. ^**a**^ Mann-Whitney was applied to determine statistical significances between the study and control groups; **^b^** Student’s* t*-test was applied to determine statistical significances between the study and control groups.

## 4. Discussion

Metals are among the chemicals of emerging concern regarding children’s health, mainly because children are more susceptible to the adverse effects of chemicals in comparison to adults due to their cognitive, physical, and physiological immaturity [5]. Children’s exposure to several metals, such as Al, As, Hg, Mn, and Pb, can cause deficits in intelligence leading to learning and neurodevelopment disorders [6,25,51,52,53]. Moreover, children who have genetic or prenatal risk factors as well as malnutrition may be more vulnerable to the adverse effects of metals [54]. In the present study, a screening for toxic and essential elements was performed on blood and hair of a children’s group from a rural area, who had learning disabilities according to their teachers. It was possible to observe an increase in the levels of some toxic metals, mainly Pb and Al, and a deficiency of the essential element selenium. Moreover, the increase of some toxic metals can be involved on lipid peroxidation and ALA-D inhibition.

With regard to cognitive ability, 37% of rural children showed low performance in the R-2 intelligence test. Moreover, most children (58%) showed visual-motor immaturity in terms of visual-motor maturation in the Bender test. Even with our sample size limitation, it was possible to observe that the poor performance in the Bender test was corroborated by the low performance in the R-2 intelligence test (Figure 1). It is known that the Bender Test has been used for detecting learning maturity, considering that a low level of perceptual motor maturation may contribute to the appearance of learning problems [41]. In other words, the frail development of cognitive functions can be a result of visual-motor immaturity. Ethier* et al.* (2012) demonstrated subclinical deficits in visual development processing in school-aged children in association with intrauterine exposure to toxic metals, such as Pb and Hg [55]. In the present study, the blood Pb levels (BLLs) were approximately 8-fold higher (~45 µg·dL^−1^) in comparison to BLLs acceptable for children in 2012 by the Centers for Disease Control and Prevention (CDC), which is 5 µg·dL^−1 ^[56] (Table 2). On the other hand, although we found levels of Pb in hair lower than the reference values, children with visual-motor immaturity had significantly higher levels of Pb in hair (~2 µg·g^−1^) when compared to children without visual motor immaturity (~1 µg·g^−1^) (Figure 2). Similar findings were found in a previous study, where low levels of Pb in children’s hair (~2 µg·g^−1^) were negatively correlated with low cognitive performance, specifically in attention function [13]. 

We believe that visual-motor immaturity found in children with higher Pb levels in hair can represent an adverse effect possibly associated with a chronic exposure to this toxic metal. Although blood is normally used for biomonitoring of toxic metals and diagnoses of the deficiency of essential elements [27] since it reflects a more recent exposure to several elements, hair however reflects past exposure or long term exposure, averaging the extent during the period of growth [13,57]. In other words, hair demonstrates the exposure over months when compared to blood that represents acute exposure [5,13,32]. However, as the guidelines for Pb blood levels were reduced for children in recent years, we think the reference values of Pb in hair for children should be reviewed, since even low levels of Pb in hair can evidence neurological effects in children and not in adults.

Additionally, we found increased levels of other two toxic metals—Ni and Al in blood and hair, respectively—comparative to recommended values (Table 2 and Table 3, respectively). A recent study with mice demonstrated nickel-induced neurologic effects after Ni oral ingestion [58]. Also, Al causes adverse effects on the central nervous system, affecting spatial learning and memory abilities [59]. Another recent study with pregnant mice exposed to Al, showed deficits in cognition and neurobehavioral functions in offspring [51]. Aluminum levels also were increased in drinking water from rural children's households (Table 4), suggesting the water as a possible source of Al contamination in the rural area, since it is known that drinking water is one of the main sources of human exposure to Al [60]. 

Moreover, essential trace element concentrations were measured in this study, since that can also cause toxicity from excessive exposures as well as health consequences due to the deficiency [60]. In the present study, although children’s nutritional status was not determined, deficiency of Se was observed in blood and hair (Table 2 and Table 3, respectively). Selenium is required for normal activity of several antioxidants enzymes, such as glutathione peroxidases (GPx). These enzymes are involved in the defense of the brain against the effects of oxidative stress since the brain is particularly vulnerable to lipid peroxidation [61]. In this respect, we suggest that selenium deficiency can be a contributor factor to lipid peroxidation observed in the present study, evidenced by MDA levels which were significantly increased in rural children when compared to urban children (Table 5). MDA is used as a biomarker of oxidative stress because it is an end-product of lipid peroxidation and its levels indicate the degree of lipid peroxidation [35]. Although we did not demonstrate association between MDA levels and metals, it is probable that lipid peroxidation is involved in the toxicological mechanism of metals in rural children. Indeed, studies have shown that Pb exposure leads to excessive production of reactive oxygen species (ROS) and changes in antioxidant defense [62]. Excessive ROS production leads to the degradation of polyunsaturated fatty acids in membrane phospholipids inducing lipid peroxidation in biologic membranes. A previous study demonstrated that MDA levels in children with high BLLs and neurological disorders were significantly increased in comparison to control groups, demonstrating that Pb promotes changes in membrane composition which leads to lipid peroxidation, and this is associated with propagation of oxidative stress [63].

Additionally, ALA-D activity was investigated in this study. No significant differences in ALA-D activity between both children’s groups were detected (Table 5). However, we observed a negative Spearman’s rank correlation between BLLs and ALA-D activity in rural children, confirming the effect of this toxic metal in ALA-D inhibition. Our findings are in agreement with another study which observed an inverse correlation between ALA-D activity and BLLs (>20 μg·dL^−1^) [37]. Lead has affinity for -SH groups and it is well known to inhibit ALA-D activity, performing as Pb exposure and effective biomarker to high exposure. Therefore, ALA-D has been used as a biomarker for the detection of lead-induced oxidative damage in red blood cells (RBCs), especially in occupational exposure [22,36,40]. However, few studies have evaluated the effects of metal on ALA-D activity in children [36]. On the other hand, the inhibition of ALA-D activity contributes to the development of oxidative stress and potential neurotoxicity due to ALA accumulation, once that it is know that ALA may be rapidly oxidized to generate ROS [63]. Moreover, the increased circulating ALA levels, which are weak gamma-aminobutyric acid (GABA) agonists, are responsible for the decrease of GABA release by presynaptic inhibition and may account for some of the behavioral disorders observed in Pb toxicity [64]. 

Lead-induced ALA-D inhibition can be reactivated* in vitro* by addition of agents such as dithiothreitol (DTT) [40]. The calculation of ALA-D reactivation index (ALA-RE) activity is considered a sensitive parameter to evaluate ALA-D inhibition [65]. Our results showed that ALA-RE was significantly higher in rural children compared to urban children (Table 5). Additionally, a positive Spearman’s rank correlation between BLLs and ALA-RE activity was observed, indicating that the ALA-D inhibition occurs due to binding of Pb to –SH groups. The increase of ALA-RE activity may be associated to an overproduction of free radicals, confirmed by an increase in MDA production [66]. 

This study has some limitations. Firstly, the small sample size is one of the most important limitations, mainly due to strict inclusion criteria applied to select the students with learning disabilities. Indeed, children from other schools of the same rural area could be included in this study. In addition, other neuropsychological tests will be used for cognitive ability evaluation of children in our future studies once the tests used in this study become qualitative tests. Secondly, another important limitation is the lack of measurements of metal levels in urban children as well as cognitive function assessment of these children. However, metal quantifications in blood and hair of urban children as well as cognitive function assessment will be investigated in future studies of our research group. Besides, as soon as the boys had short hair the collection process was hampered. Additionally, the lack of measurement of Mn levels in hair as well as Al and Hg in blood is another limitation. Despite this, studies such as ours are needed for biomonitoring of both toxic and essential trace elements, mainly in children living in potentially harmful areas, where environmental exposure to metals can be associated with adverse effects on health.

## 5. Conclusions

The present study demonstrated co-exposure to toxic metals, mainly Pb, Al and Ni, in a rural children’s group who presented learning disabilities. Additionally, deficiency of Se in these children was demonstrated. Moreover, most children presented poor performance on cognitive ability tests. The cellular damage mediated by oxidative stress is one of the pathogenic mechanisms associated with exposure to some toxic metals, such as Pb [23]. The role of oxidative stress as a toxicological mechanism induced by metals was suggested by the increase in MDA levels and ALA-D inhibition. Therefore, we suggest that it is important to identify probable sources of exposure to toxic metals to minimize the adverse effects on children’s health. Also, the evaluation of children’s nutritional status is needed by biomonitoring the levels of essential trace elements involved in important biological functions in the body, such as defense against oxidative stress.
